# Depression and Personality Traits Across Adolescence—Within-Person Analyses of a Birth Cohort

**DOI:** 10.1007/s10802-024-01188-8

**Published:** 2024-03-28

**Authors:** Ida Sund Morken, Lars Wichstrøm, Silje Steinsbekk, Kristine Rensvik Viddal

**Affiliations:** 1 Department of Psychology, NTNU – Norwegian University of Science and Technology, Trondheim, Norway; 2 Department of Child and Adolescent Psychiatry, St. Olavs University Hospital, Trondheim, Norway

**Keywords:** Major depressive disorder, Dysthymia, Structural equation modelling, Random Intercept Cross-lagged Panel Model (RI-CLPM), Adolescence, Personality traits, Predisposition model, Scar model

## Abstract

Depressive symptoms and personality traits covary in adolescents, but our understanding of the nature of this relation is limited. Whereas a predisposition explanation posits that specific personality traits increase the vulnerability for developing depression, a scar explanation proposes that depression may alter premorbid personality. Attempts to test these explanatory models have relied on analyses that conflate within-person changes and between-person differences, which limits the implications that can be drawn. Moreover, research on the early adolescent years is lacking. The present study therefore examined within-person associations between depressive symptoms and Big Five personality traits across ages 10 to 16. Children (*n* = 817; 49.9% boys) and parents from two birth cohorts in Trondheim, Norway, were assessed biennially with clinical interviews capturing symptoms of major depressive disorder and dysthymia, and self-reported Big Five personality traits. Analyses were conducted using a random intercept cross-lagged panel model, which accounts for all unmeasured time-invariant confounding effects. Increased Neuroticism predicted an increased number of depressive symptoms—and increased depressive symptoms predicted increased Neuroticism—across ages 10 to 14. Moreover, increased depressive symptoms forecast reduced Extraversion across ages 10 to 16, and reduced Conscientiousness from ages 12 to 14. Increases in Neuroticism may contribute to the development of depressive symptoms—in line with the predisposition model. As regards the scar model, depression may have an even wider impact on personality traits: increasing Neuroticism and reducing Extraversion and Conscientiousness. These effects may already be present in the earliest adolescent years.

**Supplementary Information:**

The online version contains supplementary material available at 10.1007/s10802-024-01188-8.

Depressive disorders typically manifest during adolescence (e.g., Merikangas et al., 2010) and subsequently become among the most common mental disorders (World Health Organization, 2019). Even subclinical levels of depressive symptoms are associated with current and future adverse outcomes, including impaired functioning in family, school, and peer contexts, comorbid psychiatric disorders, and suicide (Bertha & Balázs, 2013; Gibb, 2014; Rudolph & Flynn, 2014). Clearly, it is crucial to identify factors involved in the development of adolescent depression, which could inform preventative and treatment efforts.

Among such factors, personality traits have been linked with psychopathology in general, and depression in particular, even in adolescents (Klimstra et al., 2010), which is the current focus. Personality traits, as measured using the Five Factor Model (FFM: John et al., 2008), encapsulate the higher-order traits of Neuroticism, Extraversion, Conscientiousness, Agreeableness, and Openness. These traits, which are also measured in the present study, have been identified in youths as young as 10 years of age (John et al., 2008; Soto et al., 2008).

The relation between personality traits and psychopathology is considered to be complex (see, e.g., Ormel et al., 2020; Tackett, 2006). From a theoretical perspective, six explanatory models have been proposed (e.g., De Bolle et al., 2012; Tackett, 2006). First, personality traits may be a risk factor for psychopathology (i.e., the *vulnerability/predisposition* model) or influence how a given disorder manifests itself (i.e., the *pathoplasty/exacerbation* model) (e.g., Ormel et al., 2013, 2020). The first model implies that personality initiates processes that increase the risk of developing a particular disorder. For example, low scorers on Extraversion tend to experience less social support (Swickert et al., 2002), which increases the risk of depression (Rueger et al., 2016). The second model targets developmental aspects of the disorder itself, such as severity or duration (see Klein et al., 2011).

By contrast, the *scar* model posits that an existing Axis 1 disorder may cause changes in personality that persist when the depression is no longer present (De Bolle et al., 2012; Laceulle et al., 2014; Ormel et al., 2013, 2020; Tackett, 2006). A scar example may be an increased tendency to ruminate even after depression has subsided (Krause et al., 2018), and rumination is associated with higher levels of Neuroticism (Slavish et al., 2018). Notably, if an individual in remission from a major depressive episode does not return to their baselevel of, for example, Neuroticism after a certain period, the disorder may have left a scar, although not necessarily a permanent scar. Alternatively, depression may cause more temporary changes, as in the fourth, *complication* model (e.g., Ormel et al., 2020).

The two final models target third variables causing both personality traits and psychopathology, thus producing spurious correlations. The fifth model is the *spectrum/continuity* model, which implies that psychopathology and personality traits are different manifestations along the same continuum (De Bolle et al., 2012). For example, high levels of Neuroticism have been equated with depressive symptoms (Ormel et al., 2013). Closely related, although methodologically difficult to separate (Ormel et al., 2020), the sixth and final model—the *common-factor* model— highlights that psychopathology and personality traits have the same causal factors.

In the present study, we focus on the predisposition and scar explanations for the relation between depressive symptoms and the Big Five personality traits in adolescence. From a methodological standpoint, when examining longitudinal predictions as we aim to do in this study, several developmentalists recommend statistical methods that distinguish between- and within-person information (e.g., Hamaker et al., 2015; Hamaker et al., 2020; Lervåg, 2020). We argue that the predisposition and scar models are indeed within-person questions of nature (see Hamaker et al., 2020, for a conceptual discussion). Whereas *between-person* information concerns the extent to which adolescents’ personality traits covary with their depression level (e.g., if those who score high on Neuroticism also score high on depression), *within-person* information concerns whether an adolescent whose personality changes will experience a change in depression (or vice versa). Numerous scholars have highlighted the potential pitfalls of using between-person information to test hypotheses about within-person changes. This practice, known as the ecological fallacy, can lead to incorrect results because associations observed at the population level may not hold true at the individual level (Curran & Bauer, 2011). One commonly used approach in developmental research is the Cross-Lagged Panel Model (CLPM). However, the cross-lagged paths in CLPM represent a conflation of between-person and within-person information, which makes interpretation challenging (Berry & Willoughby, 2017). Moreover, because time-invariant between-person effects can confound the estimates of cross-lagged parameters, the use of CLPM to examine developmental questions, has been advised against (see, e.g., Lervåg, 2020).

Confounding effects may stem from, for example, the shared genetic vulnerability for depression and Neuroticism (e.g., Kendler et al., 2019), or trait-like parenting factors such as emotional warmth, associated with both depression (Yang et al., 2008) and Extraversion (Li et al., 2021).

In contrast, more recent statistical advancements allow the disentangling of the between- and within-person information (Usami, 2023). These methods, including the Random Intercept Cross-Lagged Panel Model (RI-CLPM: Hamaker et al., 2015) applied herein, use the participants as their own controls, thus accounting for time-invariant confounding effects (Berry & Willoughby, 2017). Evidently, however, observational studies, including those using within-person methods, do not meet all the assumptions for a causal relation (Mund & Nestler, 2019). For example, time-varying confounding remains (Berry & Willoughby, 2017). Therefore, within-person predictions may only inform on the *likelihood* of etiology and cannot be causally interpreted. Nevertheless, to advance research on the personality-depression models, within-person approaches are called for, but no such study exists.

Further, due to the significant increase in the prevalence of depression around the ages of 12 to 13 (see, e.g., Morken et al., 2020), it is important to assess potential precursors of depression before this increase occurs. However, previous studies mainly include participants from age 12. One exception is a study that followed a sample from age 10 over the course of 18 months (Zhang et al., 2020). To bridge these gaps in the literature, we therefore apply the RI-CLPM to four waves of data from a cohort that was followed biannually from ages 10 to 16. Moreover, with one exception (Goldstein et al., 2018, 2020), prior research has relied on self-report questionnaires for both depressive symptoms and personality traits, which is likely to have inflated the prospective relations between the two (Klein et al., 2011). We, however, use clinical interviews for capturing depressive symptoms and self-reports of personality traits, thus limiting the risk of common method bias.

To inform on the predisposition and scar models, which are the focus of the present study, existing prospective research—on both adolescents and adults—has applied CLPMs (e.g., Hakulinen et al., 2015; Klimstra et al., 2010). A meta-analysis of longitudinal studies on adult samples concluded that high Neuroticism, low Extraversion, and low Conscientiousness predicted depression (Hakulinen et al., 2015), which is in line with the predisposition model. The evidence indicating support for the scar model was even stronger, as depression predicted changes in all traits: higher Neuroticism and lower Extraversion, Conscientiousness, Agreeableness, and Openness (Hakulinen et al., 2015). In contrast to relatively stable rates in adulthood (Richards, 2011), the prevalence of depression changes from being infrequent in childhood to substantially increasing in adolescence (Morken et al., 2020). Moreover, adolescence is a time for considerable personality development (Soto et al., 2011). For example, Neuroticism temporarily increases in early to middle adolescence—at least in girls—and Conscientiousness and Agreeableness decline from late childhood into early adolescence, before increasing in later adolescence (Soto et al., 2011). Given these developmental changes in personality and depression, evidence in line with the predisposition and scar models in adults may not generalize to adolescents—or even *across* adolescence. Therefore, we aim to investigate reciprocal associations between depression and personality traits in adolescents while testing for developmental differences. This will be carried out in this 4-wave longitudinal study, following a cohort from ages 10 to 16.

There are fewer studies that investigate the association between depression and personality in adolescence. Some of these focus on internalizing symptoms that are measured more broadly and temperamental traits (e.g., Laceulle et al., 2014; Ormel et al., 2020). In the following, we will review studies that analyze the relation between depressive symptoms and the Big Five personality traits. To the best of our knowledge, there are seven such longitudinal studies on adolescents that are relevant for the predisposition model. All apply the CLPM approach. Whereas six of these reported that higher levels of Neuroticism predicted higher levels of depression (Calvete et al., 2016; Goldstein et al., 2020; Kercher et al., 2009; Klimstra et al., 2010; Yang et al., 2008; Zhang et al., 2020), one found that higher Neuroticism predicted *lower* levels of depression (Williams et al., 2021). With regard to Extraversion and Conscientiousness, one study chronicled adolescents with low scores on these traits to be at increased risk of depression (Klimstra et al., 2010). However, others have *not* found that Extraversion (Calvete et al., 2016; Goldstein et al., 2018; Yang et al., 2008) or Conscientiousness (Goldstein et al., 2018) predicted depression. Neither Agreeableness nor Openness have been found to predict depression (Goldstein et al., 2018; Klimstra et al., 2010). Because the Big Five personality traits have been shown to correlate (Van der Linden et al., 2010), some have partialled out the common variance by examining several traits in multivariate models, finding that only Neuroticism uniquely predicted depression (Goldstein et al., 2018; Yang et al., 2008). As such, Neuroticism should be accounted for when examining the other four traits, which will be done in the present study.

Two studies on adolescents have examined predictions from depression on the Big Five personality traits—thus informing the scar model. One of these investigated all five traits in separate models, and reported that depressive symptoms predicted higher levels of Neuroticism, and lower degrees of Extraversion, Conscientiousness, and Agreeableness (Klimstra et al., 2010). The other study only included neuroticism and found that depression predicted higher levels of this (Zhang et al., 2020).

## Current Study

This study examines the predisposition and scar explanations for the relation between depressive symptoms and the Big Five traits by—for the first time—applying a within-person approach and including participants younger than age 12. We measure symptoms of major depressive disorder (MDD) and dysthymia by means of clinical interviews in biennial follow-ups of a birth cohort sample from ages 10 to 16. To the extent that prior CLPM findings hold for within-person changes, as analyzed with RI-CLPM, we hypothesize that increased depression will predict increased neuroticism and decreased extraversion, conscientiousness, and agreeableness. Although the meta-analysis on adults found that depression predicted lower levels of openness (Hakulinen et al., 2015), the only study to investigate this in adolescents did not find this prediction (Klimstra et al., 2010). Collectively, we pose no specific scar hypotheses for openness. The present study is the first to explore potential developmental differences across adolescence in the predisposition and scar explanations.

## Methods

### Participants and Procedure

The Trondheim Early Secure Study (TESS) (Steinsbekk & Wichstrøm, 2018) comprises children from the 2003 and 2004 birth cohorts in Trondheim, Norway (*N* = 3,456). A letter of invitation, together with the Strengths and Difficulties Questionnaire (SDQ) version 4–16 (Goodman et al., 2000), was sent to the children’s homes prior to the age-4 routine health check-up. Almost all parents and their children attended the check-up (*n* = 3,358). Parents were informed orally and in writing about the TESS by a health nurse, and written consent was obtained. At age 12 the children were specifically informed about the study, and at age 16 they provided their own consent. Study procedures were approved by the Regional Committee for Medical and Health Research Ethics, Mid-Norway (approval number 2009/994).

To increase statistical power, children with emotional and behavioral problems were oversampled at baseline. To accomplish this, children were divided into four strata based on their SDQ score (0–4, 5–8, 9–11, 12–40), and the probability of being selected increased with increasing scores (37%, 48%, 70%, and 89%, from the respective strata). This oversampling was accounted for in the analyses. The drop-out rate after the provision of consent at the well-child clinic did not differ across the four SDQ strata (χ^2^(3) = 5.70, *p* = 0.127) or by sex (χ^2^(1) = 0.23, *p* = 0.973). Of the 1,250 children randomly selected for the study, 1,007 were successfully enrolled at Time 1 (*M*_*age*_ = 4.59, *SD* = 0.25; 49.1% boys) (for a flowchart of recruitment and follow-ups, see Online Supplemental Material Fig. S1). Given that our research questions pertained to explaining depression during adolescence, we included data from ages 10 (T4: *M*_*age*_ = 10.51, *SD* = 0.17), 12 (T5: *M*_*age*_ = 12.50, *SD* = 0.14), 14 (T6: *M*_*age*_ = 14.35, *SD* = 0.14), and 16 (T7: *M*_*age*_ = 16.98, *SD* = 0.31). Participants with information from at least one data wave comprised the analytical sample (*n* = 817).

Overall, attrition was unrelated to the study variables, with the exception that more symptoms of MDD (OR = 1.39, 95% CI [1.15, 1.70]) and dysthymia (OR = 1.35, 95% CI [1.12, 1.64]) at age 12 predicted attrition at age 14. Although the analyses suggested some selective attrition, they should be interpreted according to the number of attrition analyses conducted. An overall test—the Little Missing Completely at Random (MCAR) test (Little, 1988)—was therefore conducted. The results confirmed that data was not missing completely at random: χ^2^(1286.46, df = 935, *p* < 0.001). The normed test was 1.38, which is below the suggested cut-off of 2 (Ullman et al., 2001), and this indicates that data was missing at random (MAR). Demographic characteristics of the sample are presented in Table 1. Mean levels of symptoms of depression and mean levels of personality traits are presented in Table 2. The percentage of children and adolescents at each wave with x number of symptoms is presented in Online Supplemental Tables S1 and S2. Most participants (> 60%) in each wave were symptom-free, and most of those with depressive symptoms had a subclinical number of symptoms.

**Table 1:** Sample characteristics

Characteristics		%
Sex of child	Male	48.9
	Female	51.1
Sex of parent informant	Male	16.7
	Female	83.3
Parent informant	Biological parent	98.3
	Adoptive parent	1.3
	Foster parent	0.4
Biological parents’ marital status	Married	59.3
	Cohabitating > 6 months	21.9
	Cohabitating < 6 months	0.4
	Divorced/separated/no longer cohabitating	16.4
	Widowed	0.1
	Never lived together	1.9
Ethnic origin of biological mother	Norwegian	93.0
	Western Countries	2.7
	Other Countries	4.3
Ethnic origin of biological father	Norwegian	91.0
	Western Countries	5.8
	Other Countries	3.2
Informant parents’ socioeconomic status	Leader	17.5
	Professional, higher level	30.1
	Professional, lower level	30.1
	Formally skilled worker	18.5
	Farmer/fishermen	0.2
	Unskilled worker	3.6
Parent’s highest completed education	Did not complete junior high school	0.0
	Junior high school (10th grade	0.6
	Some education after junior high school	6.1
	Some collage- or university education	7.6
	Bachelor’s degree	6.2
	College degree (3–4 years study)	20.3
	Master’s degree or similar	20.3
	PhD completed or ongoing	4.4

**Table 2:** Mean Level of Symptoms of Depression and Big Five Personality Traits, Ages 10–16

Age	Mean level (SD)					
	Depression	Neuroticism	Extraversion	Conscientiousness	Agreeableness	Openness
10	1.26 (1.73)	2.56 (0.56)	3.57 (0.52)	3.61 (0.54)	4.20 (0.50)	3.77 (0.57)
12	1.36 (2.02)	2.48 (0.59)	3.64 (0.58)	3.64 (0.56)	4.20 (0.46)	3.65 (0.60)
14	1.75 (2.59)	2.50 (0.68)	3.60 (0.62)	3.59 (0.60)	4.04 (0.49)	3.48 (0.61)
16	0.50 (1.67)	2.65 (0.77)	3.33 (0.71)	3.53 (0.56)	3.95 (0.62)	3.40 (0.61)

### Measures

*Depressive symptoms* were measured as symptoms of MDD and dysthymia, as defined by the Diagnostic and Statistical Manual of Mental Disorders (DSM) and by using semi-structured psychiatric interviews. Symptoms of MDD were defined according to DSM-IV (American Psychiatric Association [APA], 1994) (the first data-wave) and DSM-5 (APA, 2013) (the following data-waves). Symptoms of dysthymia were defined according to DSM-IV in the first wave. In the last three waves, when DSM-5 had been introduced, the diagnosis Persistent Depressive Disorder (PDD) was introduced. PDD includes dysthymia, but also, for example, the possibility that MDD may be continuously present. DSM-5 allows for specification of “pure dysthymic syndrome”, and the symptoms described are the same as those described as dysthymia in DSM-4. We therefore measured the same symptoms across ages, and use the term dysthymia throughout this paper. Children and parents were interviewed separately. A symptom was considered present if it was reported to occur during the last three months by either respondent. To capture dysthymia, which should have an onset at least one year prior, we also asked for the first onset of these symptoms. The core symptoms had to be present concurrently with the other symptoms for the latter to be coded as present. At ages 10, 12, and 14, the Child and Adolescent Psychiatric Assessment (CAPA: Angold & Costello, 2000) was applied. Inter-rater reliabilities among blinded coders of 15% of audiotapes of CAPA interviews at age 10 were ICC = 0.87 for MDD symptoms and ICC = 0.85 for dysthymia symptoms. At age 16, the Schedule for Affective Disorders and Schizophrenia for School-Age Children (K-SADS: Kaufman et al., 2016) was applied. A symptom was considered present at the threshold (coded as 3), as this coincides with the DSM. Inter-rater reliabilities among blinded coders of 17% of audiotapes of K-SADS interviews were ICC = 0.81 for MDD symptoms and ICC = 0.76 for dysthymia symptoms. Symptom count scores based on the CAPA and the K-SADS were created as the sum of MDD and dysthymia symptoms.

*Personality traits* were measured by the Norwegian version of the self-reported Big Five Inventory (BFI: Soto et al., 2008), which consists of 44 items capturing Neuroticism (8 items), Extraversion (8 items), Conscientiousness (9 items), Agreeableness (9 items) and Openness (10 items). Response options range from 1 (*disagree*) to 5 (*agree*). In the present sample, the internal consistencies at ages 10, 12, 14, and 16, respectively, were as follows: Neuroticism:* α* = 0.59, 0.72, 0.81, 0.83; Extraversion: *α* = 0.54, 0.67, 0.75, 0.81; Conscientiousness: *α* = 0.65, 0.72, 0.77, 0.76; Agreeableness:* α* = 0.64, 0.71, 0.72, 0.71; Openness: *α* = 0.69, 0.74, 0.74, 0.76.

Sociodemographic information on child and parent was reported by the parent during the CAPA-interview. Sex assignment was 0 = boy and 1 = girl based on the child’s national identification number, in which the child’s biological sex at birth is registered.

### Statistical Analyses

Prospective relations were examined with a Random-Intercept Cross-Lagged Panel Model (RI-CLPM), in which within-person variance is separated from between-person variance (Hamaker et al., 2015). As regards measurement variance, the level of depression (Merikangas et al., 2010) and personality traits (Soto et al., 2011) are reported to change during adolescence. Hence, *scalar* invariance was neither envisioned nor analyzed. *Metric* invariance for depression has previously been documented in the current sample from ages 4 to 14 (Morken et al., 2020). We therefore only examined the metric invariance from ages 14 to 16, setting factor loadings to be equal over time. We also investigated metric invariance of the BFI. Given that all factors across all ages did not converge, each factor was examined separately. We applied Chen’s (2007) criteria for metric invariance (i.e., ΔCFI ≥ -0.010, ΔRMSEA ≥ 0.015, ΔSMR ≥ 0.030).

DSM-5 conceptualizes depressive disorders to be one-dimensional; thus for depression, configural invariance was not considered. A complete test of configural invariance for the Big Five across all time points did not converge. We therefore examined whether the 5-factor solution had an adequate fit at all ages, as others have found an adequate fit for the 10-year-olds, but only when adjusting for acquiescence bias (Soto et al., 2008). Therefore, we also considered the factor structure after adjusting for acquiescence bias, according to the procedure described by Soto et al. (2008).

At each wave, the observed depressive symptoms and personality trait scores were decomposed into a stable between-person part and a varying within-person part. In each model, one random intercept factor for depression and one for the personality trait in question were created, thereby capturing the participants’ overall levels of the two constructs. The factor loadings to the respective observed variables were set at 1. One latent variable was defined for each observed variable with the variance in the observed variable set to 0 and with a factor loading of 1, thereby transferring the variance to the corresponding latent variable. In effect, the latent depression and personality trait variables at each time point (*t*) capture the adolescent’s deviation from her or his own mean score across time. These latent deviations at *t* were regressed on the latent changes at *t-1*. Concurrent correlations between the error terms of these latent variables were allowed.

Because the RI-CLPM is power-demanding (e.g., Masselink et al., 2018), we examined the relation between depressive symptoms and each of the personality traits in five separate models. However, previous studies that have examined several traits in multivariate models have found that only Neuroticism uniquely predicted depression (Goldstein et al., 2018; Yang et al., 2008). We therefore examined the paths to and from Extraversion, Conscientiousness, Agreeableness, and Openness, adjusting for Neuroticism. Importantly, prior research has identified a female preponderance in depression by at least age 12 (Salk et al., 2017), and in early adolescence neuroticism increases in girls and may slightly decrease in boys (e.g., Soto et al., 2011). Because our sample size (*n* = 817) was somewhat lower than the recommended size for an RI-CLPM (Masselink et al., 2018), we were not positioned to analyze girls and boys separately.

To examine developmental effects in the predisposition and scar models, we tested whether a model where the cross-lagged paths were set to be equal across all ages fitted the data worse than a model where the paths between depression and personality traits were freely estimated, using the Satorra-Bentler Scaled Chi-square Difference Test (Satorra & Bentler, 2001). If a model in which cross-lagged paths were set to be equal did not deteriorate the model fit, we would prefer such a constrained model for parsimonious reasons. This would indicate no difference in the strength of the cross-lagged paths across age, regardless of whether the paths were statistically significant or not.

Note that in order to facilitate comparison between our results and former inquiries in which Neuroticism was not accounted for, we also estimated RI-CLPMs without this covariate. Finally, to compare our findings with previous CLPM-results—and thus inform on the impact of time-invariant confounding effects—we reran the main models using a CLPM.

All analyses were performed in Mplus 8.5, using a robust maximum likelihood estimator and population weights to correct for the oversampling of children with mental health issues. Missing data was handled using a Full Information Maximum Likelihood (FIML) procedure under the assumption that data was MAR.

## Results

Across all ages, depressive symptoms correlated positively with Neuroticism, and negatively with Extraversion, Conscientiousness, and Agreeableness (see Online Supplemental Tables S3–S17). These four traits also correlated with each other. The bivariate associations were mostly of weak effect sizes. There were, however, a few exceptions at single measurement points, and the associations between Conscientiousness and Agreeableness were positive and of moderate effect sizes across ages 10 to 14, and weak at age 16. The correlations between the random intercepts between the latent depression constructs and the five personality traits respectively were as following: Neuroticism *r* = 0.43 (*p* = 0.011), Extraversion *r* = -0.39 (*p* = 0.006), Conscientousness *r* = -0.49 (*p* = 0.009), Agreeableness* r* = 0.38 (*p* = 0.042) and Openness *r* = 0.12 (*p* = 0.545). See Online Supplemental Table S18 for the correlated time-specific residual terms. MDD evinced metric invariance, except that suicidality was more of a defining feature of MDD at age 16 than at age 14. As for dysthymia and the BFI, full metric invariance was achieved. The BFI did not demonstrate full configural invariance. As the appropriateness of a 5-factor model for 10-year-olds was of most concern, the model fit of this solution was examined with a Confirmatory Factor Analysis (CFA) at age 10. All items loaded significantly on their respective factors, except for two Extraversion items (“reserved; keeps thoughts and feelings to self” and “tends to be quiet”). Even so, the model fit was not adequate, according to fit statistics. The model fit further deteriorated when adjusting for aquiescence bias. See Supplemental Material Measurement invariance for more information, including Table S19.

### Predisposition Model

Increased Neuroticism at ages 10 and 12 forecasted an increased number of symptoms of depression two years later, at 12 and 14, respectively (Table 3). From ages 14 to 16, no effect was observed. Changes in the other four traits, adjusted for Neuroticism, were unrelated to future changes in depression (Table 3). As regards possible developmental differences, a model where the cross-lagged paths from Neuroticism to depressive symptoms were set to be equal from ages 10 to 14—whereas the path from age 14 Neuroticism to age 16 depression could vary (i.e., set to be free)—fitted the data equally well as a freely estimated model (Table 4). Thus, the relations were similar across the first two lags and differed from ages 14 to 16. For the other four personality traits, models in which cross-lagged paths from personality to depression were fixed across ages fitted the data equally well as freely estimated models (Table 4). These latter results indicate that the impact of personality traits on depression might not vary across the investigated age period. Notably, when adjusting for Neuroticism in these models, we allowed the path from Neuroticism to depression to be freely estimated from ages 14 to 16, in accordance with the model fit results in the depression-Neuroticism model.

**Table 3:** Random Intercept Cross-lagged Panel Model Analyses of Depressive Symptoms and each of the Big Five Personality Traits, ages 10–16

Standardized slope coefficients (*p*-value) B [95% CI]					
Personality traits → Depression					
	Neuroticism → Depression	Extraversion → Depression	Conscientiousness → Depression	Agreeableness → Depression	Openness → Depression
Ages 10–12	**0.15 (*p* = 0.001) [0.06, 0.24]**	-0.01 (*p* = 0.840) [-0.08, 0.06]	0.00 (*p* = 0.998) [-0.09, 0.09]	-0.03 (*p* = 0.434) [-0.09, 0.04]	0.06 (*p* = 0.143) [-0.02, 0.14]
Ages 12–14	**0.13 (*p* = 0.002) [0.05, 0.22]**	-0.01 (*p* = 0.841) [-0.07, 0.05]	0.00 (*p* = 0.998) [-0.07, 0.07]	-0.02 (*p* = 0.438) [-0.07, 0.03]	0.05 (*p* = 0.142) [-0.02, 0.11]
Ages 14–16	0.03 (*p* = 0.690) [-0.11, 0.17]	-0.01 (*p* = 0.840) [-0.12, 0.10]	0.00 (*p* = 0.998) [-0.13, 0.13]	-0.03 (*p* = 0.435) [-0.12, 0.05]	0.08 (*p* = 0.129) [-0.02, 0.18]

Depression → Personality Traits					
	Depression → Neuroticism	Depression → Extraversion	Depression → Conscientiousness	Depression → Agreeableness	Depression → Openness
Ages 10–12	**0.23 (*p* < 0.001) [0.13, 0.34]**	**-0.09 (*p* = 0.008) [-0.16, -0.03]**	-0.05 (*p* = 0.510) [-0.19, 0.10]	0.00 (*p* = 0.927) [-0.07, 0.08]	0.03 (*p* = 0.409) [-0.04, 0.09]
Ages 12–14	**0.25 (*p* < 0.001) [0.15, 0.34]**	**-0.10 (*p* = 0.009) [-0.17, -0.03]**	**-0.14 (*p* = 0.014) [-0.26, -0.03]**	0.00 (*p* = 0.928) [-0.09, 0.10]	0.03 (*p* = 0.401) [-0.04, 0.11]
Ages 14–16	0.08 (*p* = 0.247) [-0.06, 0.22]	**-0.11 (*p* = 0.009) [-0.19, -0.03]**	0.03 (*p* = 0.491) [-0.06, 0.12]	0.01 (*p* = 0.928) [-0.10, 0.11]	0.04 (*p* = 0.401) [-0.06, 0.14]

All lags between a specific personality trait and depression were set to be equal, with the following exceptions: Neuroticism predicting depression (predisposition), and depression predicting neuroticism (scar) were set to be free from ages 14 to 16. Depression predicting conscientiousness (scar) was set to be freely estimated across ages. Extraversion, conscientiousness, agreeableness and openness were controlled for the effects of neuroticism

**Table 4:** Model Comparison of Random Intercept Cross-lagged Panel Models for Depressive Symptoms and each of the Big Five Personality Traits. Testing Whether Predictions are Similar or Different across ages 10–16

	χ^2^	df	CFI	RMSEA	90% CI RMSEA	Δdf	Δχ^2^ (-p-value)
Neuroticism → depression and depression → neuroticism							
All cross-lagged free	45.11	9	0.955	0.070	0.051, 0.091		
All cross-lagged fixed vs. all free	55.05	13	0.943	0.066	0.049, 0.083	4	10.83 (0.029)
** All cross-lagged fixed except d/n14 to d/n16 vs. all free**	**44.68**	**11**	**0.958**	**0.061**	**0.043, 0.080**	**2**	**1.04 (0.594)**
Extraversion → depression and depression → extraversion							
All cross-lagged free	68.22	23	0.973	0.049	0.036, 0.063		
** All cross-lagged fixed vs. all free**	**72.24**	**27**	**0.973**	**0.045**	**0.033, 0.058**	**4**	**4.61 (0.329)**
Conscientiousness → depression and depression → conscientiousness							
All cross-lagged free	69.02	23	0.972	0.049	0.036, 0.063		
All cross-lagged fixed vs. all free	78.81	27	0.969	0.048	0.036, 0.061	4	9.77 (0.045)
** All cross-lagged fixed c → d, all free d → c**	**72.24**	**25**	**0.972**	**0.048**	**0.035, 0.061**	**2**	**5.47 (0.065)**
Agreeableness → depression and depression → agreeableness							
All cross-lagged free	60.85	23	0.975	0.045	0.031, 0.059		
** All cross-lagged fixed vs. all free**	**69.68**	**27**	**0.972**	**0.044**	**0.031, 0.057**	**4**	**9.20 (0.056)**
Openness → depression and depression → openness							
All cross-lagged free	60.28	23	0.975	0.045	0.031, 0.058		
** All cross-lagged fixed vs. free**	**63.33**	**27**	**0.976**	**0.041**	**0.028, 0.054**	**4**	**3.20 (0.526)**

Bold indicates the best fitting model for each personality trait (i.e., when the fixed model did not deteriorate the model fit of the free model, we would keep the fixed model/equal effects across ages). d = depression, n = neuroticism, c = conscientiousness

When rerunning the RI-CLPM without adjustment for Neuroticism, the results on Neuroticism were replicated (Table S20). Additionally, increased Agreeableness predicted reduced depression across ages 10 to 16 (Table S21). The results on developmental effects echoed the original RI-CLPM models (Table S21).

Finally, we reran the models with CLPM. Again, our original RI-CLPM findings on Neuroticism (Table S22) and the results on developmental effects (Table S23) were replicated. However, in the CLPM models, Openness predicted depression across all ages (Table S22).

### Scar Model

When examining scar models, increased number of depressive symptoms predicted increased levels of both Neuroticism and Conscientiousness from ages 10 to 12 and 12 to 14, whereas no effects were observed from ages 14 to 16 (Table 3). Furthermore, increases in depressive symptoms predicted reduced levels of Extraversion across all lags (Table 3). Increases in depressive symptoms were unrelated to future changes in Agreeableness and Openness (Table 3). As for developmental differences, the path from depression at age 14 on Neuroticism differed from the previous age-lags (Table 4). The paths from Conscientiousness on depression varied across ages. For the models on Extraversion, Egreeableness, and Openness in which cross-lagged panel effects were set to be equal mostly fitted the data as well as the models in which these effects were allowed to vary (i.e., indicating no developmental differences) (Table 4). Yet again, when adjusting for Neuroticism, the path from depression to Neuroticism was freely estimated from ages 14 to 16.

When we reran the RI-CLPM models without controlling for Neuroticism, the results were similar, with two exceptions. Depressive symptoms predicted reduced Agreeableness from ages 10 to 12, and reduced Conscientiousness from ages 10 to 12 (and not from ages 12 to 14, as in the original results) (Table S20). Regarding developmental differences, in addition to the 14–16 age span from depression to Neuroticism differing from previous ages, and the paths from Conscientiousness differing across ages, the paths from depression on Agreeableness also varied across all ages (Table S21).

When we reran the models with CLPM, the results echoed our original RI-CLPM results except that depression additionally predicted reduced Conscientiousness and reduced Agreeableness from ages 10 to 12 (Table S22). The path for Agreeableness from ages 10 to 12 differed from the later ages (Table S23). Thus, two apparent scar mechanisms were demonstrated with CLPM that did not appear with the RI-CLPM.

## Discussion

Although it has been long established that depressive symptoms and personality traits covary in adolescence (e.g., Klimstra et al., 2010), the understanding of this relation is limited. Two common explanations are that personality traits can pose a vulnerability for depression, and/or that depression may impact personality traits (i.e., ‘scars’). However, prior research examining these predisposition and scar explanations have all applied traditional regression-type or CLPM analyses. These approaches cannot answer whether changes in personality forecast changes in depression (or vice versa) at the level of the individual. Therefore, and for the first time, we tested the predisposition and scar explanations by applying within-person methodology in a representative community sample, using adolescents as their own controls. We identified a reciprocal relation between depressive symptoms and Neuroticism across ages 10 to 14, in line with both predisposition and scar models. Moreover, increases in depressive symptoms predicted decreased levels of Extraversion throughout ages 10 to 16, and decreased levels of Conscientiousness from ages 12 to 14, in accordance with a scar model. There were no significant within-person paths involving Agreeableness or Openness. Besides the 14–16 age span for Neuroticism, and the predictions from Conscientiousness to depression that varied across ages 10 to 16, we did not find developmental differences in the relations between depression and the personality traits.

## Reciprocal Relations Between Depression and Neuroticism

First and foremost, our results demonstrate a reciprocal within-person relation between Neuroticism and depressive symptoms across ages 10–12 and 12–14, which indicates support for both the predisposition and the scar models. This extends earlier CLPM findings to the within-person level and already from the age of 10 (Calvete et al., 2016; Goldstein et al., 2020; Kercher et al., 2009; Klimstra et al., 2010; Yang et al., 2008; Zhang et al., 2020).

Establishing reciprocal within-person predictions between depression and Neuroticism is only a first step to understanding this relation. Although our study does not inform on the mechanisms involved, prior research does allude to some possibilities. Adolescents scoring high on Neuroticism might experience more stress, which is a risk factor for depression (Ge et al., 1994). For example, Tian et al. (2019) found that the impact of high levels of Neuroticism on depression was mediated by perceived school stress. Relatedly, when faced with stress, adolescents scoring high on Neuroticism may be more prone to use emotional regulation strategies such as rumination and self-blame (Liu et al., 2020) or emotional suppression (Yoon et al., 2013). Both studies found that these strategies increased the risk for depression.

Contrary to our hypotheses, there were no significant paths between depression and Neuroticism from ages 14 to 16. This is in contrast to a CLPM study on the predisposition model in an age group comparable to ours, that *did* find that Neuroticism predicted depression (Goldstein et al., 2020). Our null-finding might be explained by the change in the depression measure from ages 14 to 16 (detailed above and in the limitations section). However, metric invariance was established. In that sense, we cannot rule out that this (null) finding is substantial. Alternatively, Neuroticism may lead to transitional increases in depressive symptoms (see, e.g., Ormel et al., 2013), or, depression might lead to temporary changes in personality (Ormel et al., 2020). Thus, a possibility is that the relation between depression and Neuroticism exists in mid-adolescence as well, but perhaps over shorter time spans than captured by our two-year lags. Changes in both depression and Neuroticism after the age 14 assessment could have receded before the age 16 assessment. Future and more intensive longitudinal within-person research could help illuminate these possibilities.

### Depressive Symptoms Predicted Reduced Extraversion and Conscientiousness

Beyond our study providing support for the scar model for Neuroticism, we also found increased number of depressive symptoms to predict reduced Extraversion. By using an within-person approach, our study extends similar findings using the CLPM (Klimstra et al., 2010). Moreover, by including participants younger than age 12, we were positioned to capture the period before the prevalence of depression increases (e.g., Morken et al., 2020). Also, we found that the predictions from depression to Extraversion were equal across ages 10 to 16, which indicates no developmental differences. It is possible that symptoms of depression (e.g., fatigue, feelings of worthlessness, loss of interest) directly curb the frequency and quality of social interactions (e.g., social withdrawal, insecurity in social settings), and for this reason forecast a decline in Extraversion. Another potential mechanism might involve erosion of social support (Coyne, 1976). Depressed individuals have been found to evoke social rejection (Segrin & Dillard, 1992)—possibly due to excessive reassurance-seeking (Starr & Davila, 2008). Thus, depressed adolescents may experience rejection when they *do* seek social support, thereby reinforcing their negative self-beliefs and further sustaining social withdrawal, as reflected in low Extraversion.

Further, we found increased depressive symptoms to forecast reduced Conscientiousness across ages 12 to 14—again in accordance with the scar model. Potential mechanisms could involve reduced executive functioning. Recent findings suggest that depression may diminish cognitive flexibility over time (Halse et al., 2022), and cognitive flexibility has been associated with lower Conscientiousness (e.g., Fleming et al., 2016). However, prospective studies are needed to determine whether an effect of depression on Conscientiousness is mediated through reduced cognitive flexibility, and why this should occur only in the 12–14 age span. Notably, however, we cannot rule out the possibility of scar-effects on Conscientiousness occurring before age 12 and after age 14, which could be more transient than this two-year measurement span could capture.

As hypothesized, neither reduced Extraversion nor Conscientiousness predicted depression. Thus, only the scar—but not the predisposition—explanation was supported for these traits. Our findings are in line with previous studies using the CLPM (Calvete et al., 2016; Goldstein et al., 2018; Yang et al., 2008), with one exception: Klimstra et al. (2010) reported that both low Extraversion and Conscientiousness predicted depression. In contrast to the current study and two of the former studies using the CLPM (Goldstein et al., 2018; Yang et al., 2008), Klimstra et al. (2010) did not control for Neuroticism. However, when we examined the models without accounting for Neuroticism, our null findings remained.

### Agreeableness and Openness

Finally, depression was not predicted by—or did not predict—changes in Agreeableness or Openness. Thus, the predisposition and scar models were not supported. Previous studies using the CLPM also indicate nonexistent reciprocal relations between depression and these traits (Goldstein et al., 2018; Klimstra et al., 2010), with one exception: Klimstra et al. (2010) found that higher levels of depression predicted lower levels of Agreeableness, yet again without accounting for Neuroticism. Interestingly, when we reran the RI-CLPMs without controlling for Neuroticism, as well as with CLPMs, depressive symptoms did predict Agreeableness—but only from ages 10 to 12. In the age groups overlapping with Klimstra et al. (2010) (age 12 and onward), we still did not find a scar-effect. It is therefore unclear why our findings diverge from those of Klimstra et al. (2010).

### Limitations

This study had a range of strengths, including a representative cohort sample, repeated assessments throughout ages 10 to 16, investigating the predisposition and scar models at the within-person level, and testing for developmental differences. Furthermore, to limit common method effects, symptoms of MDD and dysthymia were assessed by clinical interviews with both adolescents and parents, while personality traits were assessed by self-report questionnaires. Nevertheless, we acknowledge several important limitations.

First, adolescents with more depressive symptoms at age 12 more often dropped out of the study by age 14, which could potentially result in the underestimation of the increase in depressive symptoms during this period. However, considering that our prime interest was associations—and that we applied an FIML approach to missingness—we consider the possibility that factors associated with selective attrition interacted with study variables to produce the current results to be modest. Second, although we adjusted for time-invariant confounding effects, time-*varying* effects—such as stronger genetic effects in one age period than another (e.g., Kwong et al., 2021)—may still have influenced both changes in depressive symptoms and personality traits, and may therefore have produced spurious relations between them. Third, we captured depressive symptoms occurring in the prior three-month period. The three-month period in the CAPA was chosen because of concerns about the reliability of the children’s and parents’ memories over a longer time period (Angold & Costello, 2000), which was based on research showing that this reliability falls steeply after three to five months (Angold et al., 1996). Although not reported explicitly, there is probably a similar logic behind the choice of the three-month period in the K-SADS. However, this interval means that symptoms of MDD occurring between our 2-year intervals of observation may have been missed (i.e., lower validity). As such, the relation between depression and personality traits may have been underestimated, and/or null findings could represent Type II errors.

Fourth, from ages 14 to 16, we changed the clinical interview used to measure depressive symptoms from CAPA to K-SADS. The K-SADS has somewhat stricter criteria in that a depressive symptom must have been present for most of the day/at least 50% of the day—whereas in the CAPA, the symptom must have been present for at least *one hour* per day. This may have contributed to the apparent decrease in the mean number of depressive symptoms from ages 14 to 16 (Table 2)—thereby contradicting previous research that shows an *increase* in depressive symptoms during this age period (Merikangas et al., 2010). The lower mean level may have influenced the results by reducing statistical power, and thus increasing the risk of false negative cross-lagged relations from age 14 personality to depressive symptoms at age 16. Fifth, the current study focused on depressive symptoms only, although many children and adolescents have comorbid conditions such as anxiety disorders (Sharma et al., 2019) and attention-deficit/hyperactivity disorder (ADHD) (Sandstrom et al., 2021). Our study was not able to discern whether the results would, for example, differ according to different comorbidities. Relatedly, time-varying third-variable explanations for the longitudinal explanations (e.g., anxiety symptoms) cannot be ruled out.

Sixth, the moderate reliability of the BFI at age 10 is in line with research in adolescents from the USA and Canada (Soto et al., 2008). We also identified metric invariance. Complete configural invariance, however, could not be demonstrated, and adjusting for acquiescence bias (see Soto et al., 2008) did not improve the model fit. Possibly, such bias may operate differently across cultures, and/or the child’s overall intellectual capacity may be just as important. In a European sample, such as the present, Allik et al. (2004) reported that self-reports of Big Five traits (measured with a NEO inventory) were linked with intelligence among young adolescents (*n* = 2,650). This effect disappeared with increasing age. Notwithstanding, the uncertainty of our findings is somewhat higher from ages 10 to 12 than at later ages—that is, lack of associations might represent Type II errors, and the strength of the detected associations might have been underestimated.

Seventh, because the Big Five personality traits correlate (Van der Linden et al., 2010), our results might not reflect the unique contribution from each trait. Because RI-CLPM is power-demanding (Masselink et al., 2018), we were only able to adjust for the most likely candidate of confounding—Neuroticism (Goldstein et al., 2018). Eight, Norway is a country with low rates of psychiatric disorders (Bøe et al., 2021; Wichstrøm et al., 2012), and our sample may differ from contexts with overall lower and more variation in socioeconomic status and more ethnic diversity. Accordingly, the results might not be generalized to such populations, and replications in more diverse countries are needed.

Finally, we emphasize that, even though within-person approaches are endorsed in developmental psychopathology (e.g., Lervåg, 2020), there has been some criticism. Lüdtke and Robitzch (2021) proposed that a person’s temporary fluctuations around the mean (analyzed in the RI-CLPM) might offer limited insights, in contrast to the impact of factors that elucidate the variations among individuals (analyzed in the CLPM). However, this an ongoing debate, which in the years to come may further advance longitudinal analyses.

### Conclusions

This is the first within-person study—of any developmental age group—to examine the predisposition and scar explanations for the relation between depression and personality. We found evidence for within-person associations between depressive symptoms and the Big Five personality traits from age 10 onwards. The results showed reciprocal relations between depressive symptoms and Neuroticism across ages 10 to 14, which is in line with both the predisposition and scar models. Moreover, increased depressive symptoms predicted lower Extraversion across ages 10 to 16 and lower Conscientiousness from ages 12 to 14, which is in line with the scar model. The strength of the relations between depression and personality traits mostly did not vary across age. Our findings indicate that preventive and treatment efforts should consider high levels of Neuroticism as a potential vulnerability factor for depressive symptoms. Moreover, the finding that depressive symptoms may alter early adolescents’ personality traits underlines the importance of preventing depression a at this age.

### Supplementary Information

Below is the link to the electronic supplementary material.

Supplementary file1 (PDF 129 KB)

Supplementary file2 (DOCX 18 KB)

Supplementary file3 (DOCX 18 KB)

Supplementary file4 (DOCX 29 KB)

Supplementary file5 (DOCX 28 KB)

Supplementary file6 (DOCX 28 KB)

Supplementary file7 (DOCX 18 KB)

Supplementary file8 (DOCX 20 KB)

Supplementary file9 (DOCX 19 KB)

Supplementary file10 (DOCX 21 KB)

Supplementary file11 (DOCX 20 KB)

Supplementary file12 (DOCX 21 KB)

## Data Availability

Due to conditions for consent from participants, data cannot be shared.
