# Supplementary material for: Depression and Personality Traits Across Adolescence—Within-Person Analyses of a Birth Cohort
Source: Res Child Adolesc Psychopathol. 2024 Mar 28;52(8):1275–87. doi: 10.1007/s10802-024-01188-8 (PMC11289264; doi:10.1007/s10802-024-01188-8)
Supplement: Supplementary file 1 — Supplementary file1 (PDF 129 KB) [file 10802_2024_1188_MOESM1_ESM.pdf]

Figure S1

Flow chart of recruitment and follow-up

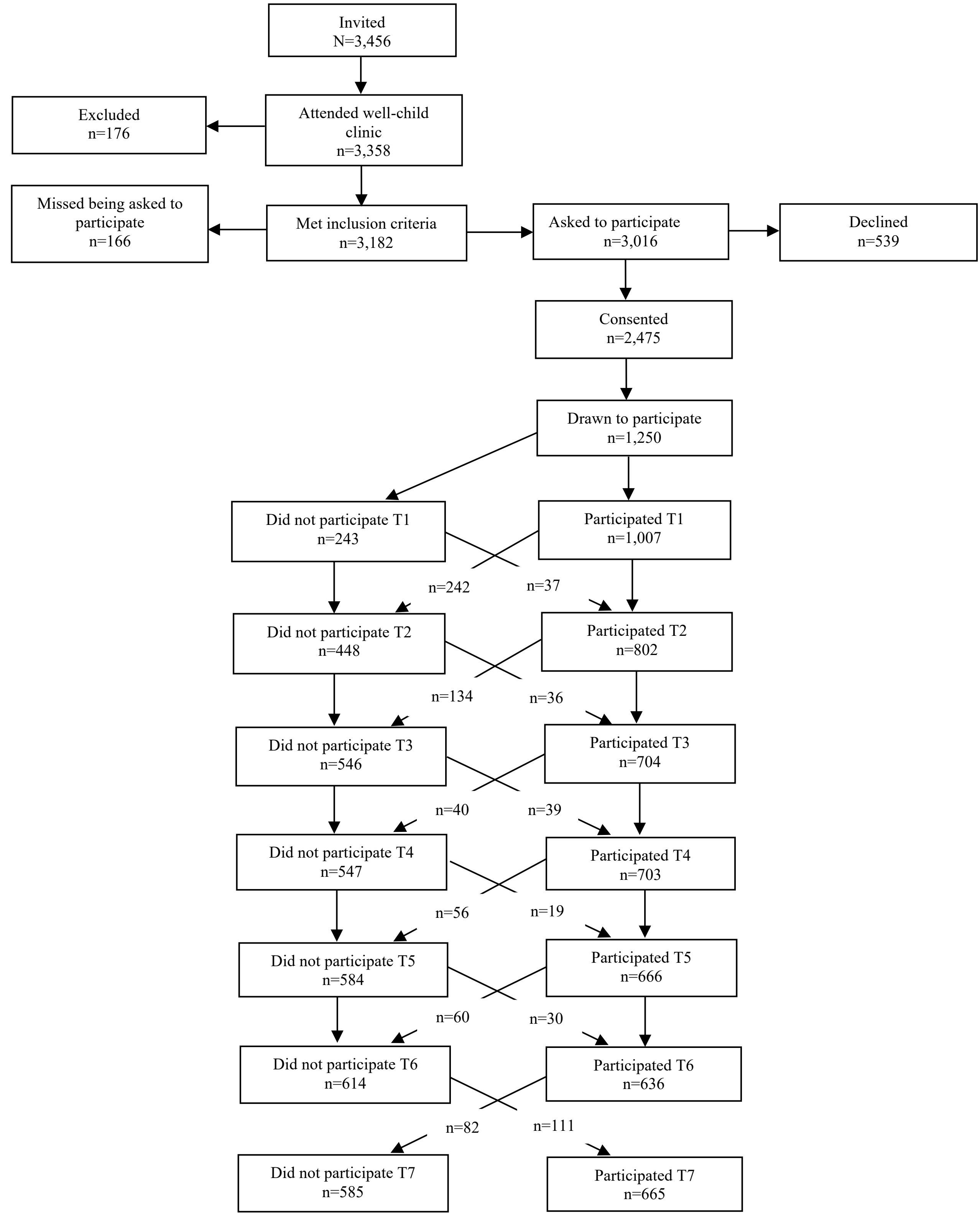

Note. Number of participants at the various assessment points is based on the number of participants invited to participate (n = 1,250) minus those who did not participate at the respective measurement point (i.e., T1, T2).
