# Supplementary material for: Depression and Personality Traits Across Adolescence—Within-Person Analyses of a Birth Cohort
Source: Res Child Adolesc Psychopathol. 2024 Mar 28;52(8):1275–87. doi: 10.1007/s10802-024-01188-8 (PMC11289264; doi:10.1007/s10802-024-01188-8)
Supplement: Supplementary file 3 — Supplementary file3 (DOCX 18 KB) [file 10802_2024_1188_MOESM3_ESM.docx]

Table S2

*Percentages with number of symptoms of Dysthymia from age 10 to age 16*

|  | Ages | | | |
| --- | --- | --- | --- | --- |
| Number of  symptoms | 10 | 12 | 14 | 16 |
| 0 | 63.5 % | 63.5 % | 61.4 % | 91.1 % |
| 1 | 24.6 % | 23.1 % | 20 % | 2 % |
| 2 | 8 % | 8.4 % | 7.1 % | 2.3 % |
| 3 | 2.7 % | 2 % | 4.1 % | 1.5 % |
| 4 | 0.5 % | 1.9 % | 4 % | 1 % |
| 5 | 0.5 % | 0.3 % | 2 % | 1.9 % |
| 6 | 0.2 % | 0.2 % | 1.2 % | 0.4 % |
| 7 | 0 % | 0.5 % | 0 % | 0.6 % |
| 8 | 0 % | 0 % | 0 % | 0.2 % |
