# Supplementary material for: Depression and Personality Traits Across Adolescence—Within-Person Analyses of a Birth Cohort
Source: Res Child Adolesc Psychopathol. 2024 Mar 28;52(8):1275–87. doi: 10.1007/s10802-024-01188-8 (PMC11289264; doi:10.1007/s10802-024-01188-8)
Supplement: Supplementary file 4 — Supplementary file4 (DOCX 29 KB) [file 10802_2024_1188_MOESM4_ESM.docx]

**Table S3**

*Pearson Product Moment Correlations between Depression and Neuroticism, ages 10-16*

|  | **1** | **2** | **3** | **4** | **5** | **6** | **7** | **8** | **9** |
| --- | --- | --- | --- | --- | --- | --- | --- | --- | --- |
| 1. Depression – age 10 | 1 |  |  |  |  |  |  |  |  |
| 2. Depression – age 12 | .35*** | 1 |  |  |  |  |  |  |  |
| 3. Depression – age 14 | .38*** | .56*** | 1 |  |  |  |  |  |  |
| 4. Depression – age 16 | .21** | .22* | .32*** | 1 |  |  |  |  |  |
| 5. Neuroticism – age 10 | .27*** | .21*** | .21*** | .03 | 1 |  |  |  |  |
| 6. Neuroticism – age 12 | .32*** | .37*** | .33*** | .12* | .45*** | 1 |  |  |  |
| 7. Neuroticism – age 14 | .37*** | .38*** | .53*** | .20*** | .36*** | .55*** | 1 |  |  |
| 8. Neuroticism – age 16 | .26*** | .30*** | .33*** | .36*** | .29*** | .42*** | .55*** | 1 |  |
| 9. Sex^a^ | .03 | .01 | .14** | .04 | -.01 | -.05 | .16** | .26*** | 1 |

*Note.* *indicates *p* <.05, ** indicates *p* <.01, *** indicates *p* <.001, ^a^0 = male; 1 = female.

**Table S4**

*Pearson Product Moment Correlations between Depression and Extraversion, ages 10-16*

|  | **1** | **2** | **3** | **4** | **5** | **6** | **7** | **8** | **9** |
| --- | --- | --- | --- | --- | --- | --- | --- | --- | --- |
| 1. Depression – age 10 | 1 |  |  |  |  |  |  |  |  |
| 2. Depression – age 12 | .35*** | 1 |  |  |  |  |  |  |  |
| 3. Depression – age 14 | .38*** | .56*** | 1 |  |  |  |  |  |  |
| 4. Depression – age 16 | .21** | .22* | .32*** | 1 |  |  |  |  |  |
| 5. Extraversion – age 10 | -.15*** | -.07 | -.09* | -.03 | 1 |  |  |  |  |
| 6. Extraversion – age 12 | -.19*** | -.20*** | -.18*** | -.16** | .48*** | 1 |  |  |  |
| 7. Extraversion – age 14 | -.23*** | -.21*** | -.28*** | -.17** | .40*** | .59*** | 1 |  |  |
| 8. Extraversion – age 16 | -.15*** | -.16*** | -.19*** | -.21*** | .38*** | .50*** | .62*** | 1 |  |
| 9. Sex^a^ | .03 | .01 | .14** | .04 | .01 | .12* | .02 | .01 | 1 |

*Note.* * indicates *p* <.05, ** indicates *p* <.01, *** indicates *p* <.001, ^a^0 = male; 1 = female.

**Table S5**

*Pearson Product Moment Correlations between Depression and Conscientiousness, ages 10-16*

|  | **1** | **2** | **3** | **4** | **5** | **6** | **7** | **8** | **9** |
| --- | --- | --- | --- | --- | --- | --- | --- | --- | --- |
| 1. Depression – age 10 | 1 |  |  |  |  |  |  |  |  |
| 2. Depression – age 12 | .35*** | 1 |  |  |  |  |  |  |  |
| 3. Depression – age 14 | .38*** | .56*** | 1 |  |  |  |  |  |  |
| 4. Depression – age 16 | .21** | .22* | .32*** | 1 |  |  |  |  |  |
| 5. Conscientiousness – age 10 | -.21*** | -.10* | -.14** | -.07 | 1 |  |  |  |  |
| 6. Conscientiousness – age 12 | -.20*** | -.23*** | -.22*** | -.20*** | .49*** | 1 |  |  |  |
| 7. Conscientiousness – age 14 | -.22*** | -.26*** | -.34*** | -.19*** | .41*** | .65*** | 1 |  |  |
| 8. Conscientiousness – age 16 | -.12* | -.17** | -.16*** | -.23*** | .30*** | .50*** | .64*** | 1 |  |
| 9. Sex^a^ | .03 | .01 | .14** | .04 | .02 | .03 | .07 | .11* | 1 |

*Note.* * indicates *p* <.05, ** indicates *p* <.01, *** indicates *p* <.001, ^a^0 = male; 1 = female.

**Table S6**

*Pearson Product Moment Correlations between Depression and Agreeableness, ages 10-16*

|  | **1** | **2** | **3** | **4** | **5** | **6** | **7** | **8** | **9** |
| --- | --- | --- | --- | --- | --- | --- | --- | --- | --- |
| 1. Depression – age 10 | 1 |  |  |  |  |  |  |  |  |
| 2. Depression – age 12 | .35*** | 1 |  |  |  |  |  |  |  |
| 3. Depression – age 14 | .38*** | .56*** | 1 |  |  |  |  |  |  |
| 4. Depression – age 16 | .21** | .22* | .32*** | 1 |  |  |  |  |  |
| 5. Agreeableness – age 10 | -.11* | -.06 | -.04 | -.05 | 1 |  |  |  |  |
| 6. Agreeableness – age 12 | -.20** | -.16*** | -.21*** | -.13* | .37*** | 1 |  |  |  |
| 7. Agreeableness – age 14 | -.14* | -.06 | -.18*** | -.15** | .36*** | .56*** | 1 |  |  |
| 8. Agreeableness – age 16 | -.12* | -.06 | -.10 | -.15** | .28*** | .43*** | .58*** | 1 |  |
| 9. Sex^a^ | .03 | .01 | .14** | .04 | .03 | .03 | .04 | .14** | 1 |

*Note.* * indicates *p* <.05, ** indicates *p* <.01, *** indicates *p* <.001, ^a^0 = male; 1 = female.

**Table S7**

*Pearson Product Moment Correlations between Depression and Openness, ages 10-16*

|  | **1** | **2** | **3** | **4** | **5** | **6** | **7** | **8** | **9** |
| --- | --- | --- | --- | --- | --- | --- | --- | --- | --- |
| 1. Depression – age 10 | 1 |  |  |  |  |  |  |  |  |
| 2. Depression – age 12 | .35*** | 1 |  |  |  |  |  |  |  |
| 3. Depression – age 14 | .38*** | .56*** | 1 |  |  |  |  |  |  |
| 4. Depression – age 16 | .21** | .22* | .32*** | 1 |  |  |  |  |  |
| 5. Openness – age 10 | .05 | .07 | .07 | .06 | 1 |  |  |  |  |
| 6. Openness – age 12 | .00 | -.07 | -.06 | .06 | .51*** | 1 |  |  |  |
| 7. Openness – age 14 | .01 | .01 | -.07 | .09 | .40*** | .58*** | 1 |  |  |
| 8. Openness – age 16 | .09 | .10 | .10 | .14* | .31*** | .38*** | .61*** | 1 |  |
| 9. Sex^a^ | .03 | .01 | .14** | .04 | .06 | .16** | -.03 | -.15** | 1 |

*Note.* * indicates *p* <.05, ** indicates *p* <.01, *** indicates *p* <.001, ^a^0 = male; 1 = female.
