# Supplementary material for: Depression and Personality Traits Across Adolescence—Within-Person Analyses of a Birth Cohort
Source: Res Child Adolesc Psychopathol. 2024 Mar 28;52(8):1275–87. doi: 10.1007/s10802-024-01188-8 (PMC11289264; doi:10.1007/s10802-024-01188-8)
Supplement: Supplementary file 5 — Supplementary file5 (DOCX 28 KB) [file 10802_2024_1188_MOESM5_ESM.docx]

**Table S8**

*Pearson Product Moment Correlations between Neuroticism and Extraversion, ages 10-16*

|  | **1** | **2** | **3** | **4** | **5** | **6** | **7** | **8** | **9** |
| --- | --- | --- | --- | --- | --- | --- | --- | --- | --- |
| 1. Neuroticism – age 10 | 1 |  |  |  |  |  |  |  |  |
| 2. Neuroticism – age 12 | .45*** | 1 |  |  |  |  |  |  |  |
| 3. Neuroticism – age 14 | .36*** | .55*** | 1 |  |  |  |  |  |  |
| 4. Neuroticism – age 16 | .29*** | .42*** | .55*** | 1 |  |  |  |  |  |
| 5. Extraversion – age 10 | -.34*** | -.16*** | -.11** | -.12** | 1 |  |  |  |  |
| 6. Extraversion – age 12 | -.24*** | -.38*** | -.20*** | -.10* | .48*** | 1 |  |  |  |
| 7. Extraversion – age 14 | -.19*** | -.23*** | -.36*** | -.17*** | .40*** | .59*** | 1 |  |  |
| 8. Extraversion – age 16 | -.16*** | -.21*** | -.19*** | -.32*** | .38*** | .50*** | .62*** | 1 |  |
| 9. Sex^a^ | -.01 | -.05 | .16** | .26*** | .01 | .12* | .02 | .01 | 1 |

*Note.* * indicates *p* <.05, ** indicates *p* <.01, *** indicates *p* <.001, ^a^0 = male; 1 = female.

**Table S9**

*Pearson Product Moment Correlations between Neuroticism and Conscientiousness, ages 10-16*

|  | **1** | **2** | **3** | **4** | **5** | **6** | **7** | **8** | **9** |
| --- | --- | --- | --- | --- | --- | --- | --- | --- | --- |
| 1. Neuroticism – age 10 | 1 |  |  |  |  |  |  |  |  |
| 2. Neuroticism – age 12 | .45*** | 1 |  |  |  |  |  |  |  |
| 3. Neuroticism – age 14 | .36*** | .55*** | 1 |  |  |  |  |  |  |
| 4. Neuroticism – age 16 | .29*** | .42*** | .55*** | 1 |  |  |  |  |  |
| 5. Conscientiousness – age 10 | -.39*** | -.23*** | -.24*** | -.10* | 1 |  |  |  |  |
| 6. Conscientiousness – age 12 | -.31*** | -.42*** | -.28*** | -.16* | .49*** | 1 |  |  |  |
| 7. Conscientiousness – age 14 | -.20*** | -.29*** | -.39*** | -.18*** | .41*** | .65*** | 1 |  |  |
| 8. Conscientiousness – age 16 | -.17*** | -.20*** | -.21*** | -.24*** | .30*** | .50*** | .64*** | 1 |  |
| 9. Sex^a^ | -.01 | -.05 | .16** | .26*** | .04 | .10 | -.02 | .09 | 1 |

*Note*. * indicates *p* <.05, ** indicates *p* <.01, *** indicates *p* <.001, ^a^0 = male; 1 = female.

**Table S10**

*Pearson Product Moment Correlations between Neuroticism and Agreeableness, ages 10-16*

|  | **1** | **2** | **3** | **4** | **5** | **6** | **7** | **8** | **9** |
| --- | --- | --- | --- | --- | --- | --- | --- | --- | --- |
| 1. Neuroticism – age 10 | 1 |  |  |  |  |  |  |  |  |
| 2. Neuroticism – age 12 | .45*** | 1 |  |  |  |  |  |  |  |
| 3. Neuroticism – age 14 | .36*** | .55*** | 1 |  |  |  |  |  |  |
| 4. Neuroticism – age 16 | .29*** | .42*** | .55*** | 1 |  |  |  |  |  |
| 5. Agreeableness – age 10 | -.36*** | -.28*** | -.16*** | -.17*** | 1 |  |  |  |  |
| 6. Agreeableness – age 12 | -.16*** | -.45*** | -.26*** | -.22*** | .37*** | 1 |  |  |  |
| 7. Agreeableness – age 14 | -.11* | -.25*** | -.33*** | -.22*** | .36*** | .56*** | 1 |  |  |
| 8. Agreeableness – age 16 | -.03 | -.12* | -.15** | -.27*** | .28*** | .43*** | .58*** | 1 |  |
| 9. Sex^a^ | -.01 | -.05 | .16** | .26*** | .09 | .06 | .02 | .10* | 1 |

*Note*. * indicates *p* <.05, ** indicates *p* <.01, *** indicates *p* <.001, ^a^0 = male; 1 = female.

**Table S11**

*Pearson Product Moment Correlations between Neuroticism and Openness, ages 10-16*

|  | **1** | **2** | **3** | **4** | **5** | **6** | **7** | **8** | **9** |
| --- | --- | --- | --- | --- | --- | --- | --- | --- | --- |
| 1. Neuroticism – age 10 | 1 |  |  |  |  |  |  |  |  |
| 2. Neuroticism – age 12 | .45*** | 1 |  |  |  |  |  |  |  |
| 3. Neuroticism – age 14 | .36*** | .55*** | 1 |  |  |  |  |  |  |
| 4. Neuroticism – age 16 | .29*** | .42*** | .55*** | 1 |  |  |  |  |  |
| 5. Openness – age 10 | -.14*** | -.07 | .01 | .09* | 1 |  |  |  |  |
| 6. Openness – age 12 | -.10* | -.17*** | -.04 | .06 | .51*** | 1 |  |  |  |
| 7. Openness – age 14 | -.05 | -.10* | -.03 | .06 | .40*** | .58*** | 1 |  |  |
| 8. Openness – age 16 | -.05 | -.06 | .01 | -.00 | .31*** | .38*** | .61*** | 1 |  |
| 9. Sex^a^ | -.01 | -.05 | .16** | .26*** | .06 | .16** | -.03 | -.15** | 1 |

*Note.* * indicates *p* <.05, ** indicates *p* <.01, *** indicates *p* <.001, ^a^0 = male; 1 = female.

**Table S12**

*Pearson Product Moment Correlations between Extraversion and Conscientiousness, ages 10-16*

|  | **1** | **2** | **3** | **4** | **5** | **6** | **7** | **8** | **9** |
| --- | --- | --- | --- | --- | --- | --- | --- | --- | --- |
| 1. Extraversion – age 10 | 1 |  |  |  |  |  |  |  |  |
| 2. Extraversion – age 12 | .48*** | 1 |  |  |  |  |  |  |  |
| 3. Extraversion – age 14 | .40*** | .59*** | 1 |  |  |  |  |  |  |
| 4. Extraversion – age 16 | .38*** | .50*** | .62*** | 1 |  |  |  |  |  |
| 5. Conscientiousness – age 10 | .32*** | .22*** | .26*** | .17*** | 1 |  |  |  |  |
| 6. Conscientiousness – age 12 | .17*** | .39*** | .29*** | .17*** | .49*** | 1 |  |  |  |
| 7. Conscientiousness – age 14 | .14*** | .24*** | .32*** | .18*** | .41*** | .65*** | 1 |  |  |
| 8. Conscientiousness – age 16 | .13** | .15*** | .17*** | .20*** | .30*** | .50*** | .64*** | 1 |  |
| 9. Sex^a^ | .01 | .12* | .02 | .01 | .04 | .10 | -.02 | .09 | 1 |

*Note.* * indicates *p* <.05, ** indicates *p* <.01, *** indicates *p* <.001, ^a^0 = male; 1 = female.
