# Supplementary material for: Depression and Personality Traits Across Adolescence—Within-Person Analyses of a Birth Cohort
Source: Res Child Adolesc Psychopathol. 2024 Mar 28;52(8):1275–87. doi: 10.1007/s10802-024-01188-8 (PMC11289264; doi:10.1007/s10802-024-01188-8)
Supplement: Supplementary file 6 — Supplementary file6 (DOCX 28 KB) [file 10802_2024_1188_MOESM6_ESM.docx]

**Table S13**

*Pearson Product Moment Correlations between Extraversion and Agreeableness, ages 10-16*

|  | **1** | **2** | **3** | **4** | **5** | **6** | **7** | **8** | **9** |
| --- | --- | --- | --- | --- | --- | --- | --- | --- | --- |
| 1. Extraversion – age 10 | 1 |  |  |  |  |  |  |  |  |
| 2. Extraversion – age 12 | .48*** | 1 |  |  |  |  |  |  |  |
| 3. Extraversion – age 14 | .40*** | .59*** | 1 |  |  |  |  |  |  |
| 4. Extraversion – age 16 | .38*** | .50*** | .62*** | 1 |  |  |  |  |  |
| 5. Agreeableness – age 10 | .32*** | .21*** | .24*** | .17*** | 1 |  |  |  |  |
| 6. Agreeableness – age 12 | .20*** | .41*** | .29*** | .20*** | .37*** | 1 |  |  |  |
| 7. Agreeableness – age 14 | .14*** | .25*** | .36*** | .20*** | .36*** | .56*** | 1 |  |  |
| 8. Agreeableness – age 16 | .07 | .19*** | .20*** | .28*** | .28*** | .43*** | .58*** | 1 |  |
| 9. Sex^a^ | .01 | .12* | .02 | .01 | .09 | .06 | .02 | .10* | 1 |

*Note.* * indicates *p* <.05, ** indicates *p* <.01, *** indicates *p* <.001, ^a^0 = male; 1 = female.

**Table S14**

*Pearson Product Moment Correlations between Extraversion and Openness, ages 10-16*

|  | **1** | **2** | **3** | **4** | **5** | **6** | **7** | **8** | **9** |
| --- | --- | --- | --- | --- | --- | --- | --- | --- | --- |
| 1. Extraversion – age 10 | 1 |  |  |  |  |  |  |  |  |
| 2. Extraversion – age 12 | .48*** | 1 |  |  |  |  |  |  |  |
| 3. Extraversion – age 14 | .40*** | .59*** | 1 |  |  |  |  |  |  |
| 4. Extraversion – age 16 | .38*** | .50*** | .62*** | 1 |  |  |  |  |  |
| 5. Openness – age 10 | .37*** | .20*** | .13** | .12** | 1 |  |  |  |  |
| 6. Openness – age 12 | .25*** | .39*** | .21*** | .13** | .51*** | 1 |  |  |  |
| 7. Openness – age 14 | .16*** | .20*** | .24*** | .13*** | .40*** | .58*** | 1 |  |  |
| 8. Openness – age 16 | .13** | .07 | .12** | .18*** | .31*** | .38*** | .61*** | 1 |  |
| 9. Sex^a^ | .01 | .12* | .02 | .01 | .06 | .16** | -.03 | -.15** | 1 |

*Note.* * indicates *p* <.05, ** indicates *p* <.01, *** indicates *p* <.001, ^a^0 = male; 1 = female.

**Table S15**

*Pearson Product Moment Correlations between Conscientiousness and Agreeableness, ages 10-16*

|  | **1** | **2** | **3** | **4** | **5** | **6** | **7** | **8** | **9** |
| --- | --- | --- | --- | --- | --- | --- | --- | --- | --- |
| 1. Conscientiousness – age 10 | 1 |  |  |  |  |  |  |  |  |
| 2. Conscientiousness – age 12 | .49*** | 1 |  |  |  |  |  |  |  |
| 3. Conscientiousness – age 14 | .41*** | .65*** | 1 |  |  |  |  |  |  |
| 4. Conscientiousness – age 16 | .30*** | .50*** | .64*** | 1 |  |  |  |  |  |
| 5. Agreeableness – age 10 | .52*** | .26*** | .28*** | .23*** | 1 |  |  |  |  |
| 6. Agreeableness – age 12 | .29*** | .51*** | .38*** | .29*** | .37*** | 1 |  |  |  |
| 7. Agreeableness – age 14 | .22*** | .31*** | .45*** | .30*** | .36*** | .56*** | 1 |  |  |
| 8. Agreeableness – age 16 | .19*** | .22*** | .26*** | .38*** | .28*** | .43*** | .58*** | 1 |  |
| 9. Sex^a^ | .04 | .10 | -.02 | .09 | .09 | .06 | .02 | .10* | 1 |

*Note*. * indicates *p* <.05, ** indicates *p* <.01, *** indicates *p* <.001, ^a^0 = male; 1 = female.

**Table S16**

*Pearson Product Moment Correlations between Conscientiousness and Openness, ages 10-16*

|  | **1** | **2** | **3** | **4** | **5** | **6** | **7** | **8** | **9** |
| --- | --- | --- | --- | --- | --- | --- | --- | --- | --- |
| 1. Conscientiousness – age 10 | 1 |  |  |  |  |  |  |  |  |
| 2. Conscientiousness – age 12 | .49*** | 1 |  |  |  |  |  |  |  |
| 3. Conscientiousness – age 14 | .41*** | .65*** | 1 |  |  |  |  |  |  |
| 4. Conscientiousness – age 16 | .30*** | .50*** | .64*** | 1 |  |  |  |  |  |
| 5. Openness – age 10 | .27*** | .06 | .00 | -.04 | 1 |  |  |  |  |
| 6. Openness – age 12 | .15*** | .23*** | .09* | .00 | .51*** | 1 |  |  |  |
| 7. Openness – age 14 | .10* | .10* | .15*** | -.03 | .40*** | .58*** | 1 |  |  |
| 8. Openness – age 16 | .03 | -.04 | -.01 | -.03 | .31*** | .38*** | .61*** | 1 |  |
| 9. Sex^a^ | .04 | .10 | -.02 | .09 | .06 | .16** | -.03 | -.15** | 1 |

*Note*. * indicates *p* <.05, ** indicates *p* <.01, *** indicates *p* <.001, ^a^0 = male; 1 = female.

**Table S17**

*Pearson Product Moment Correlations between Agreeableness and Openness, ages 10-16*

|  | **1** | **2** | **3** | **4** | **5** | **6** | **7** | **8** | **9** |
| --- | --- | --- | --- | --- | --- | --- | --- | --- | --- |
| 1. Agreeableness – age 10 | 1 |  |  |  |  |  |  |  |  |
| 2. Agreeableness – age 12 | .37*** | 1 |  |  |  |  |  |  |  |
| 3. Agreeableness – age 14 | .36*** | .56*** | 1 |  |  |  |  |  |  |
| 4. Agreeableness – age 16 | .28*** | .43*** | .58*** | 1 |  |  |  |  |  |
| 5. Openness – age 10 | .31*** | .06 | .04 | .00 | 1 |  |  |  |  |
| 6. Openness – age 12 | .17*** | .27*** | .11* | -.06 | .51*** | 1 |  |  |  |
| 7. Openness – age 14 | .07 | .10* | .10* | -.05 | .40*** | .58*** | 1 |  |  |
| 8. Openness – age 16 | .02 | -.04 | -.05 | -.03 | .31*** | .38*** | .61*** | 1 |  |
| 9. Sex^a^ | .09 | .06 | .02 | .10* | .06 | .16** | -.03 | -.15** | 1 |

*Note*. * indicates *p* <.05, ** indicates *p* <.01, *** indicates *p* <.001, ^a^0 = male; 1 = female.
