# Supplementary material for: Depression and Personality Traits Across Adolescence—Within-Person Analyses of a Birth Cohort
Source: Res Child Adolesc Psychopathol. 2024 Mar 28;52(8):1275–87. doi: 10.1007/s10802-024-01188-8 (PMC11289264; doi:10.1007/s10802-024-01188-8)
Supplement: Supplementary file 7 — Supplementary file7 (DOCX 18 KB) [file 10802_2024_1188_MOESM7_ESM.docx]

**Table S18**

*Pearson Product Moment Correlations between residuals of Depression and the Big Five personality traits, ages 10-16*

|  | Ages | | | |
| --- | --- | --- | --- | --- |
|  | 10 | 12 | 14 | 16 |
|  | Correlations between Depression and Personality traits Residuals | | | |
| Neuroticism | .21*** | .31*** | .41*** | . 31*** |
| Extraversion | -.07 | -.16* | -.21*** | -.13* |
| Conscientousness | -.13 | -.16* | -.25*** | -.15 |
| Agreeableness | -.01 | -.08 | -.13* | -.09 |
| Openness | .03 | -.10* | -.07 | .07 |

*Note.* *indicates *p* <.05, ** indicates *p* <.01, *** indicates *p* <.001.
