# Supplementary material for: Depression and Personality Traits Across Adolescence—Within-Person Analyses of a Birth Cohort
Source: Res Child Adolesc Psychopathol. 2024 Mar 28;52(8):1275–87. doi: 10.1007/s10802-024-01188-8 (PMC11289264; doi:10.1007/s10802-024-01188-8)
Supplement: Supplementary file 8 — Supplementary file8 (DOCX 20 KB) [file 10802_2024_1188_MOESM8_ESM.docx]

**Supplemental material Measurement invariance**

As an increase in depression is expected during adolescence (Merikangas et al., 2010) and there is no indication that the depression symptoms described in the DSM-5 tap into different dimensions, we considered only metric invariance. Such invariance has been documented in the current sample from age 4 to 14 (Morken et al., 2020). We therefore investigated whether metric invariance was seen between age 14 and 16, also considering that the change in the diagnostic interview from CAPA at 14 to Kiddie-SADS at 16 might have influenced the importance of individual symptoms for the depression construct. When comparing model fit, we relied on Chen’s (2007) criteria (ΔCFI < -.01, supplemented by ΔRMSEA > .015 and ΔSRMR > .030) for metric invariance. A fully constrained model did fit the MDD symptoms more poorly than a freely estimated model (Δχ2 = 108.69, Δdf = 39, p < .001). However, when symptom 9 (thoughts about death, suicidal plans or attempts) was allowed to vary between age 14 (β = .18, *p* = .01) and 16 (β = .58, *p* < .001), the constrained (invariant) model did fit the data as well as a freely estimated model (Δχ2 = 10.33, Δdf = 6, p = .112, ΔCFI = -.003, ΔRMSEA = .000, and ΔSRMR = .009). Hence, MDD evinced metric invariance, except that suicidality was more of a defining feature of MDD at age 16 than at age 14. As for dysthymia, full metric invariance was achieved (Δχ2 = 4.05, df = 6, *p* = .670, ΔCFI = -.005, ΔRMSEA = .003, and ΔSRMR = .005).

As regards BFI, a temporary disruption during adolescence of the commonly observed increasing maturity through development has often been reported (Soto et al., 2011; 2015). We did therefore not expect scalar invariance, and hence, this was not examined. A model with all factors at all time points did not converge, thus each factor was examined separately for metric invariance. The results can be found below in Supplement Table S18. Metric invariance was achieved for all dimensions except extraversion, where the CFI was above (.018) the recommended threshold (.010). When item 21 (“Tends to be quiet”) at age 10 was freely estimated invariance was achieved (B = -0.99, *p* < .001 at age 10 vs. B = -1.27, *p* < .001 at older ages).

With respect to configural invariance, a model assessing all time points did not converge. Because previous research has cast doubts about the appropriateness of the 5-factor solution fitting the responses of 10-year-olds on the BFI (Soto et al., 2008), we considered evaluating the model fit for this age group to be the most critical. Even though the model fit was not appropriate (χ2 = 2483.05, df = 892, *p* < .001, CFI = .634, TLI = .612, RMSEA = .051, 90% CI [.048-.053], and SRMR = .075), all items loaded significantly on their respective factors (*p* < .001), except for two extraversion items (item 6, *β* = -.12, *p* = .038 and item 21, *β* = -.08, *p* = .259). Prior research has found that the data of 10-year-olds are influenced by acquiescence responding (Soto et al., 2008). We therefore adjusted each item for the person’s overall tendency towards acquiescence responding on the BFI according to a procedure described by Soto et al., (2008). As it turned out, this adjustment deteriorated fit even further (χ2 = 6025.92, df = 892, p <. 001, CFI = .199, TLI = .150, RMSEA = .091, 90% CI [.089-.093], and SRMR = .060). In sum, only partial invariance was obtained: the BFI proved to have configural but not configural invariance.

**Table S19**

*Metric Invariance Comparisons between Freely Estimated and Constrained Factor Loadings*

| Factor | χ2 | | | ΔCFI | ΔRMSEA | ΔSRMR |
| --- | --- | --- | --- | --- | --- | --- |
|  | Δχ2 | Δdf | p-value |  |  |  |
| Openness | 38.01 | 27 | .078 | -.003 | -.001 | .003 |
| Agreeableness | 51.82 | 23 | .001 | -.009 | -.001 | .005 |
| Conscientiousness | 63.91 | 24 | <.001 | -.009 | .000 | .006 |
| Extraversion | 65.69 | 21 | <.001 | .018 | -.001 | -.005 |
| Extraversion item21 at age 10 set free | 0.97 | 20 | 1.00 | .008 | -.001 | .001 |
| Neuroticism | 53.01 | 21 | <.001 | -.008 | .000 | .003 |

**References**

Merikangas, K. R., He, J.-P., Burstein, M., Swanson, S. A., Avenevoli, S., Cui, L., Benjet, C., Georgiades, K., & Swendsen, J. (2010). Lifetime prevalence of mental disorders in US adolescents: results from the National Comorbidity Survey Replication–Adolescent Supplement (NCS-A). *Journal of the American Academy of Child & Adolescent Psychiatry, 49*(10), 980-989. https://doi.org/10.1016/j.jaac.2010.05.017

Morken, I. S., Viddal, K. R., Ranum, B., & Wichstrøm, L. (2020). Depression from preschool to adolescence—five faces of stability. *Journal of Child Psychology and Psychiatry*, *62*(8), 1000-1009. <https://doi.org/10.1111/jcpp.13362>

Soto, C. J., John, O. P., Gosling, S. D., & Potter, J. (2008). The developmental psychometrics of big five self-reports: acquiescence, factor structure, coherence, and differentiation from ages 10 to 20. *Journal of Personality and Social Psychology, 94*(4), 718-737. <https://doi.org/10.1037/0022-3514.94.4.718>

Soto, C. J., John, O. P., Gosling, S. D., & Potter, J. (2011). Age differences in personality traits from 10 to 65: Big Five domains and facets in a large cross-sectional sample. *Journal of Personality and Social Psychology, 100*(2), 330-348. <https://doi.org/10.1037/a0021717>

Soto, C. J., & Tackett, J. L. (2015). Personality traits in childhood and adolescence: Structure, development, and outcomes. *Current Directions in Psychological Science, 24*(5), 358-362. <https://doi.org/10.1177/0963721415589345>
