# Supplementary material for: Depression and Personality Traits Across Adolescence—Within-Person Analyses of a Birth Cohort
Source: Res Child Adolesc Psychopathol. 2024 Mar 28;52(8):1275–87. doi: 10.1007/s10802-024-01188-8 (PMC11289264; doi:10.1007/s10802-024-01188-8)
Supplement: Supplementary file 9 — Supplementary file9 (DOCX 19 KB) [file 10802_2024_1188_MOESM9_ESM.docx]

**Table S20**

*Random Intercept Cross-lagged Panel Model Analyses of Depressive Symptoms and each of the Big Five Personality Traits, ages 10-16*

| Standardized slope coefficients (*p*-value) B [95% CI] | | | | | |
| --- | --- | --- | --- | --- | --- |
| Personality traits 🡪 Depression | | | | | |
|  | Neuroticism 🡪  Depression | Extraversion 🡪  Depression | Conscientiousness 🡪 Depression | Agreeableness 🡪 Depression | Openness 🡪  Depression |
| Ages 10-12 | **.15 (*p*=.002) [.06, .24]** | -.03 (*p*=.372) [-.09, .06] | -.05 (*p*=.256) [-.13, .03] | **-.08 (*p*=.024) [-.16, -.01]** | .03 (*p*=.498) [-.05, .10] |
| Ages 12-14 | **.13 (*p*=.002) [.05, .21]** | -.03 (*p*=.372) [-.08, .03] | -.04 (*p*=.256) [-.11, .03] | **-.07 (*p*=.024) [-.12, -.01]** | .02 (*p*=.498) [-.04, .08] |
| Ages 14-16 | .03 (*p*=.685) [-.11, .17] | -.05 (*p*=.372) [-.15, .06] | -.07 (*p*=.256) [-.19, .05] | **-.11 (*p*=.024) [-.20, -.02]** | .04 (*p*=.498) [-.07, .14] |
| Depression 🡪 Personality Traits | | | | | |
|  | Depression 🡪  Neuroticism | Depression 🡪  Extraversion | Depression 🡪  Conscientiousness | Depression 🡪  Agreeableness | Depression 🡪  Openness |
| Ages 10-12 | **.23 (*p*<.001) [.13, .34]** | **-.07 (*p*=.027) [-.13, -.01]** | -.10 (*p*=.161) [-.24, .03] | **-.17 (*p*=.022) [-.33, -.02]** | .02 (*p*=.605) [-.04, .07] |
| Ages 12-14 | **.26 (*p<*.001) [.15, .34]** | **-.08 (*p*=.027) [-.15, -.01]** | **-.15 (*p*=.009) [-.27, -.04]** | .02 (*p*=.709) [-.08, .17] | .02 (*p*=.605) [-.05, .09] |
| Ages 14-16 | .08 (*p*=.245) [-.06, .22] | **-.08 (*p*=.027) [-.16, -.01]** | .07 (*p*=.156) [-.02, .15] | -.01 (*p*=.911) [-.12, .12] | .02 (*p*=.605) [-.07, .11] |

*Note.* All lags between a specific personality trait and depression were set to be equal, with the following exceptions: Neuroticism predicting depression (predisposition), and depression predicting neuroticism (scar) were set to be free from ages 14 to 16, and depression predicting conscientiousness (scar) and agreeableness (scar) were set to be freely estimated across all ages.
