# Supplementary material for: Depression and Personality Traits Across Adolescence—Within-Person Analyses of a Birth Cohort
Source: Res Child Adolesc Psychopathol. 2024 Mar 28;52(8):1275–87. doi: 10.1007/s10802-024-01188-8 (PMC11289264; doi:10.1007/s10802-024-01188-8)
Supplement: Supplementary file 11 — Supplementary file11 (DOCX 20 KB) [file 10802_2024_1188_MOESM11_ESM.docx]

**Table S22**

*Cross-Lagged Panel Model Analyses of Depressive Symptoms and the Big Five Personality Traits, ages 10-16*

| Standardized coefficients (*p*-value) B [95% CI] | | | | | |
| --- | --- | --- | --- | --- | --- |
| Personality traits 🡪 Depression | | | | | |
|  | Neuroticism 🡪  Depression | Extraversion 🡪  Depression | Conscientiousness 🡪 Depression | Agreeableness 🡪 Depression | Openness 🡪  Depression |
| Ages 10-12 | **.15 (*p<*.001) [.09, .21]** | -.04 (*p*=.144) [-.08, .01] | -.04 (*p*=.069) [-.09, .00] | -.06 (*p*=.054) [-.11, .00] | **.06 (*p*=.008) [.06, .40]** |
| Ages 12-14 | **.12 (*p<*.001) [.07, .18]** | -.03 (*p*=.143) [-.07, .01] | -.03 (*p*=.071) [-.07, .00] | -.04 (*p*=.053) [-.09, .00] | **.05 (*p*=.008) [.06, .40]** |
| Ages 14-16 | .04 (*p*=.502) [-.08, .16] | -.05 (*p*=.127) [-.11, .01] | -.06 (*p*=.070) [-.12, .00] | -.07 (*p*=.051) [-.14, .00] | **.08 (*p*=.005) [.02, .14]** |
| Depression 🡪 Personality Traits | | | | | |
|  | Depression 🡪  Neuroticism | Depression 🡪  Extraversion | Depression 🡪  Conscientiousness | Depression 🡪  Agreeableness | Depression 🡪  Openness |
| Ages 10-12 | **.21 (*p*<.001) [.14, .27]** | **-.09 (*p<*.001) [-.12, -.04]** | **-.10 (*p<*.001) [-.15, -.05]** | **-.13 (*p*=.015) [-.23, -.03]** | .04 (*p*=.134) [-.00, .03] |
| Ages 12-14 | **.22 (*p<*.001) [.16, .28]** | **-.09 (*p<*.001) [-.14, -.04]** | **-.11 (*p<*.001) [-.17, -.05]** | .03 (*p*=.340) [-.03, .10] | .04 (*p*=.123) [-.00, .03] |
| Ages 14-16 | .04 (*p*=.498) [-.08, .15] | **-.10 (*p<*.001) [-.15, -.44]** | .06 (*p*=.115) [-.01, .14] | .04 (*p*=.342) [-.04, .12] | .05 (*p*=.126) [-.02, .12] |

*Note*. All lags between a specific personality trait and depression were set to be equal, with the following exceptions: Neuroticism predicting depression (predisposition), and depression predicting neuroticism (scar) were set to be free from ages 14 to 16, depression predicting conscientiousness (scar) were set to be free from ages 14 to 16, and depression predicting agreeableness (scar) were set to be free from ages 10 to 12.
