# Supplementary material for: Depression and Personality Traits Across Adolescence—Within-Person Analyses of a Birth Cohort
Source: Res Child Adolesc Psychopathol. 2024 Mar 28;52(8):1275–87. doi: 10.1007/s10802-024-01188-8 (PMC11289264; doi:10.1007/s10802-024-01188-8)
Supplement: Supplementary file 12 — Supplementary file12 (DOCX 21 KB) [file 10802_2024_1188_MOESM12_ESM.docx]

**Table S23**

*Model Comparison of Cross-lagged Panel Models for Depressive Symptoms and each of the Big Five Personality Traits. Testing Whether Predictions are Similar or Different across ages 10-16*

|  | χ^2^ | df | CFI | RMSEA | 90% CI RMSEA | Δdf | Δχ^2^ (-p-value) |
| --- | --- | --- | --- | --- | --- | --- | --- |
| Neuroticism 🡪 depression and depression 🡪 neuroticism |  |  |  |  |  |  |  |
| All cross-lagged free | 57.48 | 12 | .950 | .068 | .051, .086 |  |  |
| All cross-lagged fixed  vs. all cross-lagged free | 78.80 | 16 | .918 | .069 | .054, .085 | 4 | 21.41 (<.001) |
| **All cross-lagged fixed except d/n14 to d/n16 vs. all free** | **58.84** | **14** | **.942** | **.063** | **.047, .080** | **2** | **0.82 (.663)** |
| Extraversion 🡪 depression and depression 🡪 extraversion |  |  |  |  |  |  |  |
| All cross-lagged free | 115.84 | 29 | .945 | .061 | .049, .072 |  |  |
| **All cross-lagged fixed**  **vs. all free** | **121.22** | **33** | **.944** | **.057** | **.046, .068** | **4** | **5.80 (.214)** |
| Conscientiousness 🡪 depression and depression 🡪 conscientiousness |  |  |  |  |  |  |  |
| All cross-lagged free | 95.21 | 29 | .958 | .053 | .041, .065 |  |  |
| All cross-lagged fixed  vs. all free | 110.11 | 33 | .951 | .053 | .043, .065 | 4 | 15.01 (.005) |
| **All cross-lagged fixed except d14 to c16 vs. all free** | **99.18** | **32** | **.957** | **.051** | **.040, .062** | **3** | **3.47 (0.324)** |
| Agreeableness 🡪 depression and depression 🡪 agreeableness |  |  |  |  |  |  |  |
| All cross-lagged free | 111.52 | 29 | .941 | .059 | .048, .071 |  |  |
| All cross-lagged fixed  vs. all free | 123.58 | 33 | .935 | .058 | .047, .069 | 4 | 12.63 (.013) |
| **All cross-lagged fixed except d4 to a5 vs. all free** | **113.51** | **32** | **.942** | **.055** | **.045, .067** | **3** | **3.77 (0.288)** |
| Openness 🡪 depression and depression 🡪 openness |  |  |  |  |  |  |  |
| All cross-lagged free | 87.74 | 29 | .959 | .050 | .038, .062 |  |  |
| **All cross-lagged fixed**  **vs. all free** | **91.57** | **33** | **.959** | **.047** | **.035, .058** | **4** | **3,75 (.441)** |

*Note*. Bold indicates the best fitting model for each personality trait (i.e., when the fixed model did not deteriorate the model fit of the free model, we would keep the fixed model/equal effects across ages). d=depression, n=neuroticism, c=conscientiousness, a=agreeableness.
